# Breaking Bad News: A Valid Concern among Clinicians

**Published:** 2019-07

**Authors:** Gelareh Biazar, Kourosh Delpasand, Farnoush Farzi, Abbas Sedighinejad, Ali Mirmansouri, Zahra Atrkarroushan

**Affiliations:** 1 Anesthesiology Research Center, Department of Anesthesiology, Alzahra Hospital, Guilan University of Medical Sciences, Rasht, Iran.; 2 Department of Medical Ethics, Guilan University of Medical Sciences, Rasht, Iran.; 3 Department of Statistics, Guilan University of Medical Sciences, Rasht, Iran.

**Keywords:** *Breaking Bad News*, *Clinicians*, *Guilan*, *Patients*, *Way*

## Abstract

**Objective:** Delivering bad news is the duty of specialist physicians. However, they find it very difficult due to insufficient experience. In this study, the way faculty and residents of Guilan University of Medical Sciences (GUMS) delivered bad news to the patients was investigated.

**Method** **:** This study was conducted at hospitals affiliated to GUMS during 2017. A questionnaire containing 18 items on environmental and psychical support was filled through a face to face interview. The ﬁrst 10 questions evaluated psychical support and the next eight environmental supports. The scoring of each question ranged from 10 to 50, with 10 indicating “never” and 50 “always”.

**Results: **According to the analysis of 235 questionnaires, only 32 (13.6%) of the participants had been taught to deliver bad news and 195(83%) felt they need educational courses. Also, 40 (17%) believed that they had enough ability to deliver these massages. No significant differences were observed among physicians who had taken teaching courses in breaking bad news to patients.

**Conclusion: **This study revealed that educational courses to improve physicians’ communication skill to break bad news to patients are strongly warranted.

Bad news is defined as “any information that adversely and negatively affects the patients’ view of future” (1). When a patient receives bad news, his/her life changes (2).The way these massages are delivered is highly important because the lack of sufficient skill and knowledge can negatively impact both the patient and physician (3). Physicians find this situation complex and stressful (4). Breaking bad news has psychological effects on both patient and doctor (5). Studies have demonstrated patients’ need and interest to know the truth (6, 7). Therefore, if they feel that their doctor is not honest, it makes them more anxious and damages their trust (8, 9). The reasons that prevents the doctors from being truthful about breaking bad news include fear of being blamed, unexpected evoking reactions by the patients and their family, and expressing piteous emotions and questions (10-13). It is demonstrated that telling the truth has several benefits, such as strengthening the physician-patient relationship, less complains against the physician and better decision making for the treatment process (14).

However, high-risk situations, such as the probability of suicide or harm to others, are an exception. In spite of the benefits of being truthful, if bad news is not delivered appropriately, it will have negative consequences (15).

 Several published articles have evaluated the efficacy of different protocols to improve the quality of physician-patient’ communications. However, it still remains as the most daunting duty of physician. In addition, the findings of these studies could not be generalized to our society, as several factors, including, culture, beliefs, and religion, family relationship, and community, potentially influence the physician-patient relationship (16). For example, the patients’ right to know the truth and the physicians’ duty to tell the truth, which is a leitmotif in Western countries, might be problematic in the Middle East (17-19). 

A few similar studies have been conducted on this subject in Iran, but not in Guilan province. 

It seems that the issue has not been appropriately addressed among our physicians. Thus, due to the importance of a culture-based protocol for breaking bad news, the health care team in each area must follow their localized guidelines (4). In this research, as the first steps toward development of practical approaches to deliver bad news, the way physicians in Guilan academic hospitals managed the issue was investigated.

## Materials and Methods

This study was conducted at teaching hospitals affiliated to Guilan University of Medical Sciences (GUMS), Rasht Iran, during 2017. Firstly, the aim and the method of the research were explained to the faculty and residents of GUMS. If they accepted to participate, a questionnaire was filled through a face to face interview. This was a multicenter study and a specialist was responsible for data collection in each hospital. Finally, all the gathered questionnaires were delivered to the authorized specialist. 

***Ethics***

The study protocol was approved by the Research Ethics Committee of the GUMS (Ref: IR.GUMS.REC.1396.243) and participants provided written informed consent.

***Questionnaire***

In this paper, a questionnaire, taken from the study of Ghaffarinejad et al with confirmed validity and reliability, was used (20). This 18-item questionnaire had 2 main parts. The ﬁrst 10 questions evaluated psychical support and 8 items evaluated environmental support. Each question was scored from 10 to 50, with the score of 10 indicating “Never” and 50 “Always”. Baseline information was gathered and the need for implementing specific educational courses and the history of attendance at these classes were also questioned. Finally, data were included in statistical analysis.

***Sample Size***

According to a preliminary study on 20 faculty and residents, in which 80% declared that they had insufficient knowledge to break bad news to the patients, the sample size of 235 was determined. 

## Results

Of the 243 returned questionnaires, eight were not completed, so 235 questionnaires were analyzed. Of the responders, 97 (41.3%) were specialists and 138(58.7%) residents. Of the participants, 111 (47.2%) were female and 124 (52.8%) were male. The mean years of experience was 8.97±7.14, with the least years of experience being one and the most 27 years. Only 32 (13.6%) of the participants had been taught to deliver bad news and 195(83%) felt they need a course to develop this skill. However, 40 (17%) believed they had enough ability to deliver such news. The mean scores for each item are shown in Tables 1 and 2. Moreover, only 19.1%of the physicians believed that it is the patients’ right to know about their exact survival time. A significant difference was found in gender and environmental & psychical support scores, which indicated that females achieved higher scores. (P = 0.001). Also, a positive correlation was found between degree and psychical support score, as it was significantly superior among specialists compared to residents (P = 0.001). However, this difference was not observed in environmental support score (p = 0.18).

The correlation between gender and degree (specialist or resident) with achieved scores are presented in Tables 3.

## Discussion

Irrespective of physicians’ specialty, they are frequently faced with a default condition to break bad news, and the quality of breaking such news directly affects patients’ health outcomes (9). Studies have shown a significant difference between patients and physicians’ preferences on the ways to break bad news. In addition, these studies have indicated that most clinicians found this task a complex communication skill and they had much difficulty to tell the truth to their patients, so they strongly felt the need for training on this important issue (10, 12, 19 and 21). Aein et al in Shahre Kord, Iran, in a research article demonstrated the importance of the proper way to deliver bad news. Mothers of children with cancer were interviewed, and most of them were dissatisfied about the way they received the bad news (22). A study was conducted by Arbabi et al at the Cancer Institute of Tehran University of Medical Sciences, and it found that only 8% of physicians had been taught to disclose bad news (23). In a similar study performed by Ghaffarinejad et al, faculty members and residents of academic hospitals in Kerman were enrolled, and it was found that only one of them had passed an educational course for breaking bad news. However, no significant difference was found between faculty and residents in communication skill in that study (20). Jalali et al in Birjand, Iran, investigated the experiences of patients and their families who had received bad news by health care providers and different reactions were reported. They declared that more attention should be paid to this topic. Moreover, supportive approaches must be established when breaking bad news (24). The questionnaire used in this study only expressed the way that bad news were delivered but did not discover participants’ experience or ability. However, in line with previous studies, the results of this study revealed the need for developing educational courses for breaking bad news. In this study, 86.4% of the physicians had not been trained to break bad news appropriately and 83% declared the necessity of these courses. This seems to be a promising finding because it indicates that only 3.4% had neither been under teaching interventions nor felt its importance. In this study, no significant difference was reported between trained and non-trained groups, demonstrating that the current educational programs are not efficient and should be modified. Another significant finding was that a small percent of our participants (11.5%) always believed in the necessity of being truthful to patients and deliver bad news as soon as possible. No significant differences were observed regarding the ways of giving bad news between physicians who had teaching programs with others. Specialists significantly achieved higher scores, according to psychical support items. It shows that our clinicians have got some experience and do not follow a standard guideline to communicate with their patients. These findings uncover the fact that current educational strategies might not be sufficient enough to improve the physician-patient communication skills. This problem is not limited to Iran, even in developed countries, such as USA and UK, a few studies have assessed patient-based evidence for the recommended protocols on this issue (25). Monden K from Baylor University at Dallas, USA, reported that 91% of the physicians believed that giving bad news is an important skill, but only 40% of them had received training for it (12). On the whole, similar to the most academic centers in Iran, there is not a well-planned teaching protocol for breaking bad news. The problem is partly due to the fact that, historically, medical students’ tendency has been toward focusing on technical proficiency rather than concentrating on the importance of proper communication skills (4, 12). Given the mentioned deficiency in the current literature, it is time to oppose the traditional teaching approaches in which medical students just follow the specialists’ behavior in clinical setting and everything goes on according to some experience instead of formulized protocols. This topic should play a more prominent role in teaching curriculums of medical schools (9). We acknowledge that professionalism and interpersonal communication skills are subjective and difficult to be assessed by a questionnaire (26), however, the obtained results are undeniable and revealed that the current educational programs should be modified and communication skill training courses must be added to the curriculum of medical schools.

This survey is valuable because it tried to shed light on the importance of a subject that has not received sufficient attention. Also, this was a multicenter study with a proper sample size. Guilan University, with several academic hospitals that constitutes the majority of residential specialty fields, can report valuable results.

**Table 1:** The Environmental Support Questions and the Prevalence of the Answers among Faculty & Residents of GUMS

		**Never**	**Seldom**	**Sometimes**	**Often**	**Always**
1	I attract the family support	1(0.4%)	10(4.3%)	27(11.5%)	94(40%)	103(43. 8%)
2	I appraise the patients information requirement	1(0.4%)	13(5.5%)	23(9.8%)	95(40.4%)	103(43. 8%)
3	I give them an exact survival	2(0.9%)	32(13.6%)	79(33.6%)	77(32.8%)	45(19.1%)
4	I hold their arm for warm empathy	27(11.5%)	73(31.1%)	68(28.9%)	49(20.9%)	18(7.7%)
5	I highlight the importance of the issue before telling the details	2(0.9%)	6(2.6%)	37(15.7%)	98(41.7%)	92(39.1%)
6	I also carry them hope	2(0.9%)	16(.6.8%)	42(17.2%)	74(31.5%)	101(43%)
7	I exactly tell them how long they will live	86(33.6%)	87(37%)	43(18.3%)	16(6.8%)	3(1.3%)
8	I care about their concerns and interests	7(3%)	86(2.6%)	54(23%)	88(37.4%)	80(34%)
9	I deliver bad news as soon as they are aware from their illness	19(8.1%)	39(16.6%)	84(35.7%)	66(28.1%)	27(11.5%)
10	I encourage them to express their feeling	8(3.4%)	37(14.7%)	73(31.1%)	80(34%)	37(15.7%)

GUMS: Guilan University of Medical Sciences

**Table 2:** The Psychical Support Questions and the Prevalence of the Answers among Faculty & Residents of GUMS

		**Never**	**Seldom**	**Sometimes**	**Often**	**Always**
1	I choose a private location	2(0.9%)	15(6.4%)	21(8.9%)	116(49.4%)	81(34.5%)
2	I choose a time that relatives feel comfortable	3(1.3%)	9(3.8%)	30(12.8%)	113(48.1%)	80(34%)
3	I sit beside them not at my table	13(5.5%)	39(16.6%)	83(35.3%)	62(29%)	38(16.2%)
4	I wear my medicine coat	9(3.8%)	26(11.1%)	39(16.6%)	84(35.7%)	77(32.8%)
5	I introduce them to patient support groups	23(9.8%)	44(18.7%)	62(26.4%)	66(28.1%)	40(17%)
6	I make sure that a relative is available	2(0.9%)	11(4.7%)	49(20.6%)	87(37%)	86(36.6%)
7	I ask secretor to hold my phone calls	12(6.55.1%)	21(8.9%)	40(17%)	74(31.5%)	88(37.4%)
8	I Switch of turn of my cellphone and pager	11(4.7%)	30(12.8%)	36(15.3%)	74(31.5%)	84(35.7%)

GUMS: Guilan University of Medical Sciences

**Table 3:** The Correlation between Physicians’ Gender and Degree with Environmental & Psychical Support Scores

	**Gender**	**Number**	**X ± SD**		**Degree**	**Number**	**X ± SD**	
Environment al Support Score	Male	124	28.74± 5.76	t=4.93 p=0.001	Specialist	97	30.82±4.79	t=1.33 p=0.18
	Female	111	31.99± 4.06		Resident	138	29.89±5.58	
Psychical Support Score	Male	124	35.25±4.11	t=3.88 p=0.001	Specialist	97	37.60±4.027	t=4.86 p=0.001
	Female	111	37.20± 3.51		Resident	138	35.17± 3.59	

A significant difference was found regarding gender and environmental support score (P = 0.005). Moreover, a positive correlation was found between degree and psychical support score (P = 0.001).

## Limitation

This study lacked information on patients’ satisfaction with the current approaches. Also, as this was not an objective study, it only showed physicians’ point of view; however, they do not necessarily act as they believe. Furthermore, in this study, only academic hospitals were evaluated and physicians’ performance in private wards was not investigated.

## Conclusion

Because of limited experience, the majority of faculty and residents of GUMS are faced by several difficulties and fears when breaking bad news to patients or families. The present study strongly highlighted the need for more practical interventions to improve this essential skill. Also, further well-planned studies are required to find all deficiencies in this area.
